# The Effect of the 10-Valent Pneumococcal Nontypeable Haemophilus influenzae Protein D Conjugate Vaccine on H. influenzae in Healthy Carriers and Middle Ear Infections in Iceland

**DOI:** 10.1128/JCM.00116-19

**Published:** 2019-06-25

**Authors:** Hildigunnur Sveinsdóttir, Jana Birta Björnsdóttir, Helga Erlendsdóttir, Martha Á. Hjálmarsdóttir, Birgir Hrafnkelsson, Ásgeir Haraldsson, Karl G. Kristinsson, Gunnsteinn Haraldsson

**Affiliations:** a University of Iceland, Faculty of Medicine, Reykjavík, Iceland; b Department of Clinical Microbiology, Landspitali University Hospital, Reykjavík, Iceland; c BioMedical Centre, University of Iceland, Reykjavík, Iceland; d Department of Mathematics, University of Iceland, Reykjavík, Iceland; e Children’s Hospital Iceland, Reykjavík, Iceland; University of Iowa College of Medicine

**Keywords:** *Haemophilus influenzae*, Iceland, carriage, epidemiology, otitis media, protein D, vaccines

## Abstract

Vaccinations with the 10-valent pneumococcal conjugated vaccine (PHiD-CV) started in Iceland in 2011. Protein D (PD) from H. influenzae, which is coded for by the *hpd* gene, is used as a conjugate in the vaccine and may provide protection against PD-positive H. influenzae. We aimed to evaluate the effect of PHiD-CV vaccination on H. influenzae in children, both in carriage and in acute otitis media (AOM). H. influenzae was isolated from nasopharyngeal swabs collected from healthy children attending 15 day care centers in 2009 and from 2012 to 2017 and from middle ear (ME) samples from children with AOM collected from 2012 to 2017. All isolates were identified using PCR for the *hpd* and *fucK* genes. Of the 3,600 samples collected from healthy children, 2,465 were culture positive for H. influenzae (68.5% carriage rate); of these, 151 (6.1%) contained *hpd*-negative isolates. Of the 2,847 ME samples collected, 889 (31.2%) were culture positive for H. influenzae; of these, 71 (8.0%) were *hpd* negative. Despite the same practice throughout the study, the annual number of ME samples reduced from 660 in 2012 to 330 in 2017. The proportions of *hpd*-negative isolates in unvaccinated versus vaccinated children were 5.6% and 7.0%, respectively, in healthy carriers, and 5.4% and 7.8%, respectively, in ME samples. The proportion of *hpd*-negative isolates increased with time in ME samples but not in healthy carriers. The number of ME samples from children with AOM decreased. The PHiD-CV had no effect on the proportion of the *hpd* gene in H. influenzae from carriage, but there was an increase in *hpd*-negative H. influenzae in otitis media. The proportions of *hpd*-negative isolates remained similar in vaccinated and unvaccinated children.

## Abstract (precis)

Vaccinations with the 10-valent pneumococcal conjugated vaccine (PHiD-CV) started in Iceland in 2011. Protein D (PD) from H. influenzae, which is coded for by the *hpd* gene, is used as a conjugate in the vaccine and may provide protection against PD-positive H. influenzae.

## INTRODUCTION

Haemophilus influenzae is a common colonizer of the upper respiratory tracts of healthy individuals, especially children. However, H. influenzae can cause various infections in the respiratory tract and connected body sites, especially in young children (1).

A conjugated vaccine targeting H. influenzae type b (Hib) has almost eradicated invasive diseases caused by H. influenzae in countries where the vaccine is widely used. Before vaccination, serotype b caused up to 96% of all H. influenzae invasive diseases (2). Nowadays, the most common H. influenzae infections are respiratory tract infections, e.g., otitis media caused by nonencapsulated strains (NTHi). NTHi are the most common cause of acute otitis media (AOM) after pneumococci (3).

In Iceland, Hib vaccine has been in included in the national immunization program since 1989 (4), but the 10-valent pneumococcal conjugated vaccine (PHiD-CV; Synflorix) was introduced into the program in April 2011 in a 2-plus-1 schedule without catch-up. No other protein-conjugated pneumococcal vaccine had previously been included (5). Protein D (PD) from NTHi is used as the conjugate for 8 of the 10 pneumococcal serotypes in PHiD-CV (6). PD is a highly conserved outer membrane protein found in most, but not all, H. influenzae strains (7). The inclusion of PD in the vaccine may provide protection in young children against infections caused by PD-positive H. influenzae (8).

The aim of this study was to evaluate the effect of vaccination with the PHiD-CV on H. influenzae carriage in healthy children attending day care centers (DCC) and from the middle ears (MEs) of children with AOM.

## MATERIALS AND METHODS

### Sampling and isolation.

Nasopharyngeal swabs were collected from healthy children 1 to <7 years of age attending 15 DCCs in the greater Reykjavík area in March 2009 and from 2012 to 2017, as previously described (5). When H. influenzae isolates showing different colony morphologies were seen from DCC samples, isolates of each respective form were included in the study.

All ME samples from children <7 years of age with suspected otitis media that were submitted to the Department of Clinical Microbiology, Landspitali University Hospital, in 2009 to 2017 as previously described were included (5). Samples were usually collected with a swab from the outer ear after a rupture in the tympanic membrane following AOM or if discharge was secreted through a tympanostomy tube. When two or more H. influenzae isolates were identified from ME samples from the same patient within 30 days, only the first isolate was included in the study.

All samples were selectively cultured for H. influenzae using chocolate blood agar with bacitracin discs and incubated in 5% CO_2_ overnight.

### Identification.

The identification of all isolates was confirmed using PCR for the *hpd* gene, which codes for PD. Isolates negative with the hpd#1 primers were further tested with the hpd#3 primers to increase sensitivity, and isolates negative with both *hpd* primers were confirmed as H. influenzae using PCR for the *fucK* gene, which codes for fuculose kinase, using two sets of primers to increase sensitivity (9–11). The identification of isolates negative for both genes was confirmed as non-H. influenzae using a matrix-assisted laser desorption ionization–time of flight (MALDI-TOF) system.

In addition, all isolates were tested for the presence of capsular genes by using PCR for the *bexA* gene, and all positive isolates were further typed using type-specific primers (12, 13). All primers used in the study are listed in Table S1 in the supplemental material. Some of the primers and the PCRs were slightly modified for endpoint PCR.

### Statistical analysis.

The primary service area for the Landspitali University Hospital was considered to be within 100 km driving distance from the hospital. To correct the number of ME samples with regard to the population, the population demographic information for children <7 years of age in this region was obtained from Statistics Iceland (www.statice.is).

All children born before 31 December 2010 were grouped as unvaccinated, and children born 1 January 2011 or later were grouped as vaccinated.

GraphPad QuickCalcs (GraphPad Software, San Diego, CA) two-sided Fisher’s exact test was used to compare groups. A likelihood ratio test was used to test the time trend in the number of ME samples, carriage rates, and proportions of *hpd*-negative isolates per year during the sampling period. All levels of significance were set at 0.05.

### Ethics.

Informed consent was obtained from parents of all children in the DCC studies. The study was approved by The National Bioethics Committee (VSNb2013010015/03.07), the appropriate authorities at the Landspitali University Hospital, and the DCC’s directors.

## RESULTS

### Bacterial isolates.

In total, 3,600 nasopharyngeal swab samples were collected from healthy children (aged 1 to 6 years; median age, 4.3), of which, 2,465 samples were culture positive for H. influenzae (68.5% overall carriage rate). Carriage rates fluctuated between years but did not change with relation to time, although it was highest in 2009 (81.6%) and lowest in 2017 (57.1%). The carriage rate was highest in children less than 2 years of age and gradually declined with age (see Table S2 in the supplemental material) but did not change with relation to vaccination status (see Table S3). Of the 2,465 nasopharyngeal swab samples positive for H. influenzae, 114 contained H. influenzae showing two types of colony morphology. In these cases, both isolates were tested for the presence of the *hpd* and *bexA* genes. Of the 2,465 nasopharyngeal swab samples positive for H. influenzae, 151 (6.1%) contained H. influenzae isolates that were negative in the *hpd* PCR but positive in *fucK* PCR. The numbers of isolates from each year are listed in Table 1.

**TABLE 1:** Samples from the nasopharynx of healthy children

Category	2009	2012	2013	2014	2015	2016	2017	Total
No. of samples	520	465	471	566	533	539	506	3,600
Samples from vaccinated children (no. [%])	0 (0)	0 (0)	18 (3.8)	126 (22.3)	252 (47.3)	377 (69.8)	506 (100)	1,279 (35.5)
Samples positive for H. influenzae (no. [% carriage])	421 (81.6)	282 (60.6)	339 (72.0)	355 (62.7)	395 (74.1)	384 (71.1)	289 (57.1)	2,465 (68.5)
Samples containing *hpd*-negative isolates (no. [%])	27 (6.4)	14 (5.0)	16 (4.7)	21 (5.9)	24 (6.1)	27 (7.0)	22 (7.6)	151 (6.1)
No. of samples containing *bexA*-positive isolates	3	5	0	5	9	0	6	28
Type(s) (*n*)	b (2), e (1)	e (2), f (3)		f (5)	e (2), f (7)		e (1), f (5)	b (2), e (6), f (20)

Of the 114 nasopharyngeal swab samples containing two types of colony morphology, 15 samples (13.1%) contained both *hpd*-positive and -negative isolates, and 5 samples (4.4%) contained both capsulated isolates and NTHi.

The numbers of ME samples received for culture at the Department of Microbiology remained stable for the years 2009 to 2011, but these samples were not stored and were therefore not included in the study.

During 2012 to 2017, 2,847 ME samples from children (aged 0 to 6 years; median age, 1.7 years) were collected, of which, 889 (31.2%) were positive for H. influenzae. The highest number of ME samples was collected in 2013 (*n* = 683), and then the number of ME samples gradually declined during the period (*P* < 0.001) and was lowest in 2017 (*n* = 330). The proportion of ME samples culture positive for H. influenzae was highest in 2012 (36.1%) but lowest in 2016 (23.7%). The numbers of samples and the proportions of culture-positive samples were highest in children less than 2 years of age and gradually declined with age (Table S2). The numbers of ME samples from vaccinated children reduced with time as expected (Table S3). Of the 889 ME samples culture positive for H. influenzae, 64 (7.2%) were negative in the *hpd* PCR but positive in *fucK* PCR. The numbers of isolates from each year are listed in Table 2.

**TABLE 2:** Samples from ME of children <7 years of age

Category	2009	2010	2011	2012	2013	2014	2015	2016	2017	Total since 2012
No. of samples	725	664	717	660	683	492	336	346	330	2,847
Samples from vaccinated children (no. [%])				225 (34.1)	487 (71.3)	421 (85.6)	296 (88.0)	340 (98.3)	325 (98.5)	2,094 (73.6)
Population under 7 years of age in the uptake area (*n*)				25,918	26,030	26,134	25,899	25,274	24,711	
Samples positive for H. influenzae (no. [%])	239^*a*^ (33.0)	233^*a*^ (35.1)	238^*a*^ (33.2)	235 (35.6)	230 (33.7)	169 (34.3)	80 (23.8)	82 (23.7)	93 (28.2)	889 (31.2)
Samples containing *hpd*-negative isolates (no [%])				12 (5.1)	13 (5.7)	12 (7.1)	5 (6.3)	9 (11.0)	13 (14.0)	64 (7.2)
No. of samples containing *bexA*-positive isolates				1	0	1	2	0	0	4
Type(s) (*n*)				e		f	b, e			b (1), e (2), f (1)

a Isolates were not stored and were therefore not included in the study.

During the first half of the study period, 2012 to 2014, the number of ME samples was 1,835 from an average of 26,027 children in the uptake area (annual incidence rate of 23.5 cases/1,000 children). During the second half of the study period, 2015 to 2017, the number of ME samples was 1,012 from an average of 25,294 children (annual incidence rate of 13.3 cases/1,000 children) (*P* < 0.001). The proportion of culture-positive ME samples decreased from 34.6% during 2012 to 2014 (649/1,835) to 25.2% (262/1,011) during 2015 to 2017 (*P* < 0.001), and the annual incidence rate of culture-positive ME samples was reduced from 8.1/1,000 children to 3.4/1,000 children, respectively.

No difference was seen in the proportions of samples containing *hpd*-negative isolates with relation to time in healthy carriers, but an increase in the proportion of *hpd*-negative isolates was seen in ME samples (*P* < 0.005). However, no difference was seen in the proportions of *hpd*-negative isolates in unvaccinated children versus vaccinated children, in either sample group (Table 3).

**TABLE 3:** Numbers of *hpd*-positive and -negative samples from unvaccinated and vaccinated children in each sample group

Isolate	DCC samples			ME samples		
	No. (%) from unvaccinated children	No. (%) from vaccinated children	*P* value	No. (%) from unvaccinated children	No. (%) from vaccinated children	*P* value
*hpd* negative	88 (5.6)	63 (7.0)	0.163	12 (5.4)	52 (7.8)	0.294
*hpd* positive	1,482 (94.4)	832 (93.0)		210 (94.6)	615 (92.2)	
Total	1,570 (100)	895 (100)		222 (100)	667 (100)	

The proportions of encapsulated strains in both sample groups were low, with type f being the most common serotype (Tables 1 and 2). Three isolates of H. influenzae type b were found, two in the nasopharynx of healthy boys, 4.8 and 6.2 years old, attending the same DCC in 2009, and one isolate was found in an ME sample from a 9-month-old boy in 2015.

## DISCUSSION

In this study, we investigated almost 3,400 isolates of H. influenzae for the presence of the *hpd* gene, and we showed that most of the isolates had the gene. If vaccination with PD induces immune response against PD-positive H. influenzae, the proportion of PD-negative isolates would likely increase over time in vaccinated children, both in carriage and in disease. We saw an increase in the proportion of *hpd*-negative isolates over time in ME samples but not in samples from healthy carriers. However, the percentages of isolates lacking the gene were similar in unvaccinated and vaccinated children, both in carriage and in disease.

Vaccination with the PHiD-CV started in Iceland in 2011, and the vaccine uptake was high; >97% received at least two doses (14). The first fully vaccinated children entered the DCCs in autumn 2012 and were sampled for the first time in 2013. In 2017, all children in the DCCs were fully vaccinated. Since the introduction of the PHiD-CV, we have only seen minor fluctuation in the carriage rate of H. influenzae in Iceland, which is similar to other studies (15). The carriage rate of H. influenzae in Iceland remained high during the study and did not seem to change with relation to the vaccine, although the lowest rate was found in 2017. The carriage rate of pneumococci in the same sampling group was also lowest in 2017 (5). In Brazil, carriage rates of NTHi increased after vaccination with PHiD-CV and protection against NTHi was not observed (16). Carriage rates of H. influenzae seem to differ largely in various studies, and the carriage rate in Iceland is considerably higher than was found in most other studies (16–20), possibly because of the different climate and/or different time period when samples were collected; however, the lowest carriage rate was found to be similar to that in Holland (15).

Before 2012, the total number of ME samples and the proportion of H. influenzae-positive samples remained similar. A significant reduction of ME samples was seen after the vaccination with PHiD-CV, and in addition, the proportion of H. influenzae-positive ME samples was also reduced significantly. Similar results were seen with the pneumococci: the proportion of samples positive for pneumococci from middle ear samples decreased significantly (5). It is possible that the PD conjugate vaccine provides protection against AOM infections caused by H. influenzae (21). However, it is also possible that the decrease in sampling and the isolation of H. influenzae from ME infections in children are a combination of a direct effect from the PD, which is used as a conjugate in the vaccine, and reduced pneumococcal ME infections after vaccination (5, 22).

The vast majority of the H. influenzae isolates, both from carriage and ME samples, were NTHi. The most common serotype was serotype f, as seen in other studies (15, 19). Although Hib has almost disappeared from invasive infections (2), we found two isolates of H. influenzae type b in healthy carriers and one in an ME sample. It is therefore clear that despite high uptake of Hib vaccine (23), Hib can still be found in low numbers in the community.

The main weakness of the study is having isolates from only 1 year prior to the start of the PHiD-CV vaccination. However, the inclusion of almost 3,400 isolates, almost 1,800 isolates thereof from unvaccinated children, and the testing all isolates for the presence of the *hpd* gene can be considered strengths in the study.

Considering the global burden of respiratory diseases and repeated exacerbations among patients, it is important to monitor possible changes of H. influenzae in healthy carriers and in disease. The role of NTHi in respiratory diseases justifies the need for a vaccine in order to reduce repeated use of antibiotics.

### Conclusions.

The PHiD-CV had no effect on the prevalence of the *hpd* gene in H. influenzae from healthy carriers, but there was an increase in *hpd*-negative H. influenzae in otitis media. The proportions of isolates lacking the *hpd* gene remained similar in unvaccinated and vaccinated children. However, the number of ME samples and the proportion of H. influenzae-positive samples from children with AOM submitted to the Department of Clinical Microbiology decreased.

## Supplementary Material

Supplemental file 1
