# Supplementary material for: The Effect of the 10-Valent Pneumococcal Nontypeable Haemophilus influenzae Protein D Conjugate Vaccine on H. influenzae in Healthy Carriers and Middle Ear Infections in Iceland
Source: J Clin Microbiol. 2019 Jun 25;57(7):e00116-19. doi: 10.1128/JCM.00116-19 (PMC6595461; doi:10.1128/JCM.00116-19)
Supplement: Supplemental file 1 [file JCM.00116-19-s0001.pdf]

Supplementary table S1. Primers used in the study.

Primer	Note	Nucleotide sequence (5'-3')	Target	Reference
*hpd#*1-f	a	GATTGGAAAGAAACACAAGAAAAAG	*hpd* (PD)	(1, 2)
*hpd#*1-r	a	CACCATCGGCATATTTAACCA		
hpd#3f		GGTTAAATATGCCGATGGTGTTG		
hpd#3r		TGCATCTTTACGCACGGTGTA		
*fucK*1-f	a	CACTTTCGGCGTGGATGG	*fucK* (fuculose kinase)	(2, 3)
*fucK*1-r		AAGATTTCCCAGGTGCCAGA		
*fucK*2-f		ATGGCGGGAACATCAATGA		
*fucK*2-r		ACGCATAGGAGGGAAATGGTT		
Hi 1	a	CGTTTGTATGATGTTGATCC	*bex*A (capsule)	(4)
Hi 2	a	CCATGTCTTCAAAATGATG		
AcsA/f	a	GCAACCATCTTACAACTTAG	type a	(5)
AcsA/r	a	CGGTGTCCTGTGTTTAG		
b1		GCGAAAGTGAACTCTTATCTC	type b	(4)
b2	a	CTTACGCTTCTATCTCGGT		
c1		TCTGTGTAGATGATGGTTCA	type c	(4)
c4	b	TCAATGAAAGTAACCCATTC		This study
d1	a	TGATGACCGATACAACCTG	type d	(4)
d2	a	TCCACTCTTCAAACCATTC		
EcsC2-F	c	CACACTACCTTTTGAGAAGAG	type e	This study
EcsC-R	a	TAGTTTGAAAGAACCCTCTG		(5)
FcsA-f	a	CCTGAAATTTGCTATTACTTTA	type f	(5)
FcsA-r	a	GTGGTCTATTTCCATTCTCTT		

a Primers shortened from original publication to adjust melting temperatures and to adapt to endpoint PCR
b A new primer designed for this study, Accession number CP031241
c A new primer designed for this study, Accession number HM053635
All primers were modified and designed using the on-line Primer3 program
(http://biotools.umassmed.edu/bioapps/primer3_www.cgi)

References:

1. Wang X, Mair R, Hatcher C, Theodore MJ, Edmond K, Wu HM, Harcourt BH, Carvalho Mda G, Pimenta F, Nymadawa P, Altantsetseg D, Kirsch M, Satola SW, Cohn A, Messonnier NE, Mayer LW. 2011. Detection of bacterial pathogens in Mongolia meningitis surveillance with a new real-time PCR assay to detect *Haemophilus influenzae*. Int J Med Microbiol 301:303-9.
2. Meyler KL, Meehan M, Bennett D, Cunney R, Cafferkey M. 2012. Development of a diagnostic real-time polymerase chain reaction assay for the detection of invasive *Haemophilus influenzae* in clinical samples. Diagn Microbiol Infect Dis 74:356-62.
3. Binks MJ, Temple B, Kirkham LA, Wiertsema SP, Dunne EM, Richmond PC, Marsh RL, Leach AJ, Smith-Vaughan HC. 2012. Molecular surveillance of true nontypeable *Haemophilus influenzae*: an evaluation of PCR screening assays. PLoS One 7:e34083.
4. Falla TJ, Crook DW, Brophy LN, Maskell D, Kroll JS, Moxon ER. 1994. PCR for capsular typing of *Haemophilus influenzae*. J Clin Microbiol 32:2382-6.
5. Wroblewski D, Halse TA, Hayes J, Kohlerschmidt D, Musser KA. 2013. Utilization of a real-time PCR approach for *Haemophilus influenzae* serotype determination as an alternative to the slide agglutination test. Mol Cell Probes 27:86-9.

Supplementary table S2. Number of samples and culture positive samples with regards to age in A: the nasopharynx of healthy children, B: ME samples.

A

	<2 years	2-3 years	4-6 years
Number of samples*	115	1470	2011
Culture positive samples	105	1131	1226
% culture positive	91.3	76.9	61.0
hpd negative	1	74	76
% *hpd* negative	1.0	7.0	6.2

* the age of 4 children was unknown, 3 of them were culture positive

B

	<2 years	2-3 years	4-6 years
Number of samples	1725	837	285
Culture positive samples	605	251	33
% culture positive	35.1	30.0	11.6
hpd negative	45	17	2
% *hpd* negative	7.4	6.8	6.1

Supplementary table S3. Culture positive and negative samples with respect to children's vaccination status in A: the nasopharynx of healthy children, B: ME samples.

A

	2009	2012	2013	2014	2015	2016	2017
Samples from unvaccinated children	520	465	453	440	281	164	
Culture negative	100	184	130	178	94	68	
Culture positive	420	281	323	262	187	96	
% culture positive	81.4	60.4	71.3	59.5	66.5	58.5	
Samples from vaccinated children			18	126	252	375	506
Culture negative			2	33	45	87	217
Culture positive			16	93	207	288	289
% culture positive			88.9	73.8	82.1	76.6	57.1

B

	2012	2013	2014	2015	2016	2017
Samples from unvaccinated children	435	196	71	40	6	5
Culture negative	280	145	58	38	6	4
Culture positive	155	51	13	2		1
% culture positive	35.6	26.0	18.3	5.0	0.0	20.0
Samples from vaccinated children	225	487	421	296	340	325
Culture negative	144	307	267	218	258	233
Culture positive	81	180	154	78	82	92
% culture positive	36.0	37.0	36.6	26.4	24.1	28.3
